# Supplementary figures and images for: Cyclodipeptides from Pseudomonas aeruginosa modulate the maize (Zea mays L.) root system and promote S6 ribosomal protein kinase activation
Source: PeerJ. 2019 Aug 28;7:e7494. doi: 10.7717/peerj.7494 (PMC6717507; doi:10.7717/peerj.7494)

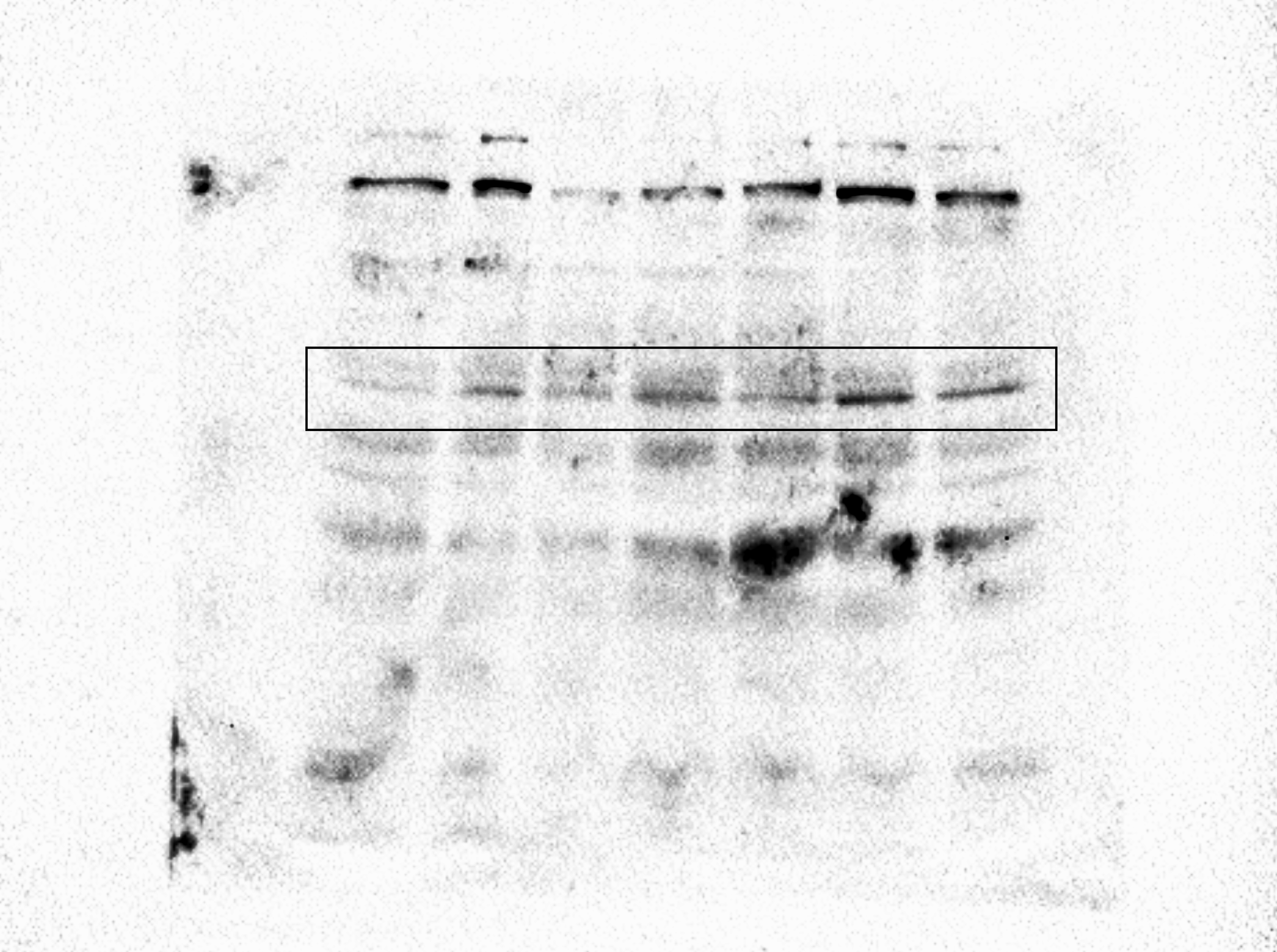

Supplement: Supplemental Information 1 — The complete membrane from which the upper panel of Fig. 5A was taken. [file peerj-07-7494-s001.png]

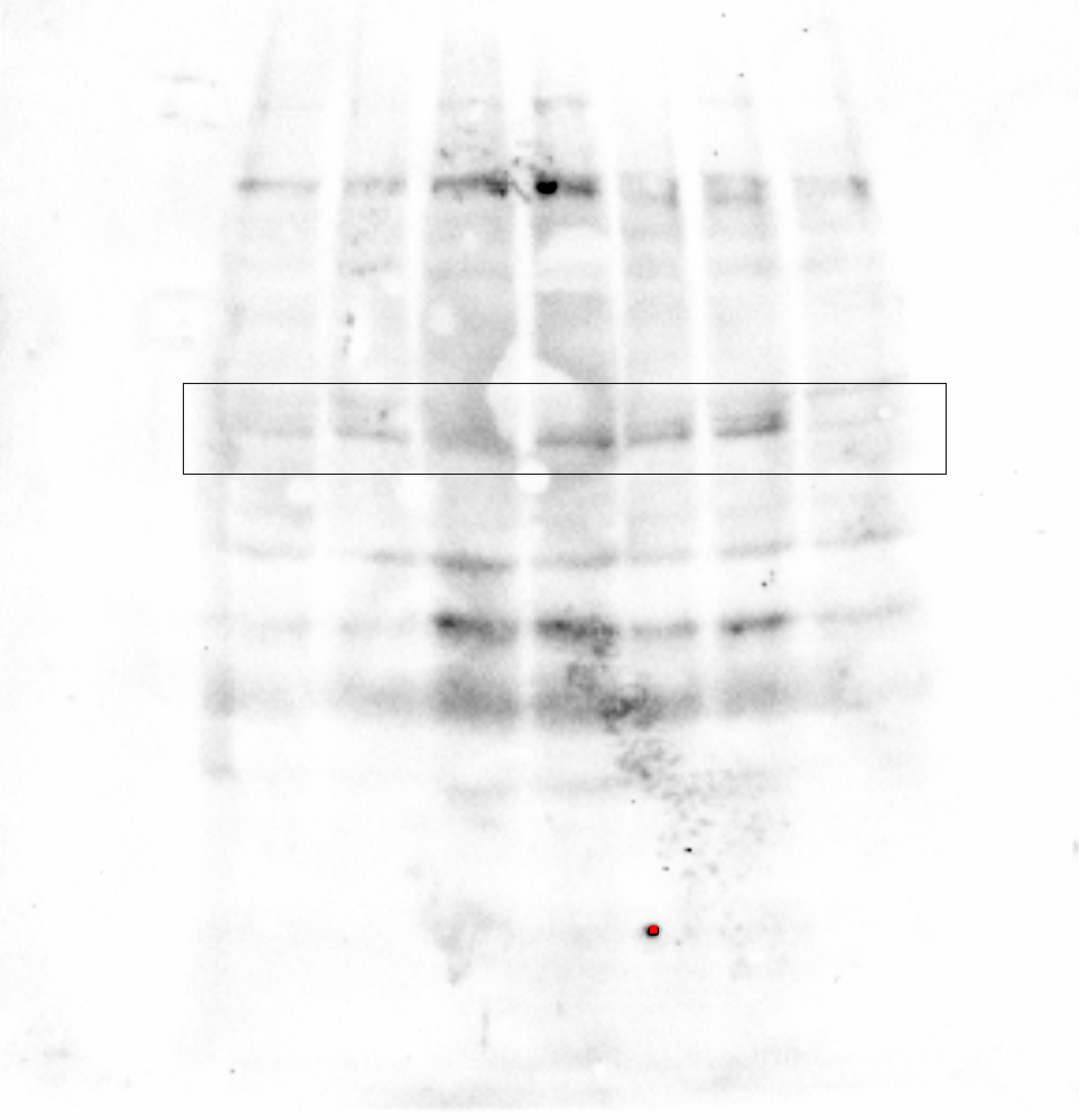

Supplement: Supplemental Information 2 — The second membrane resulting from an independent experiment presented in the upper panel of Fig. 5. [file peerj-07-7494-s002.png]

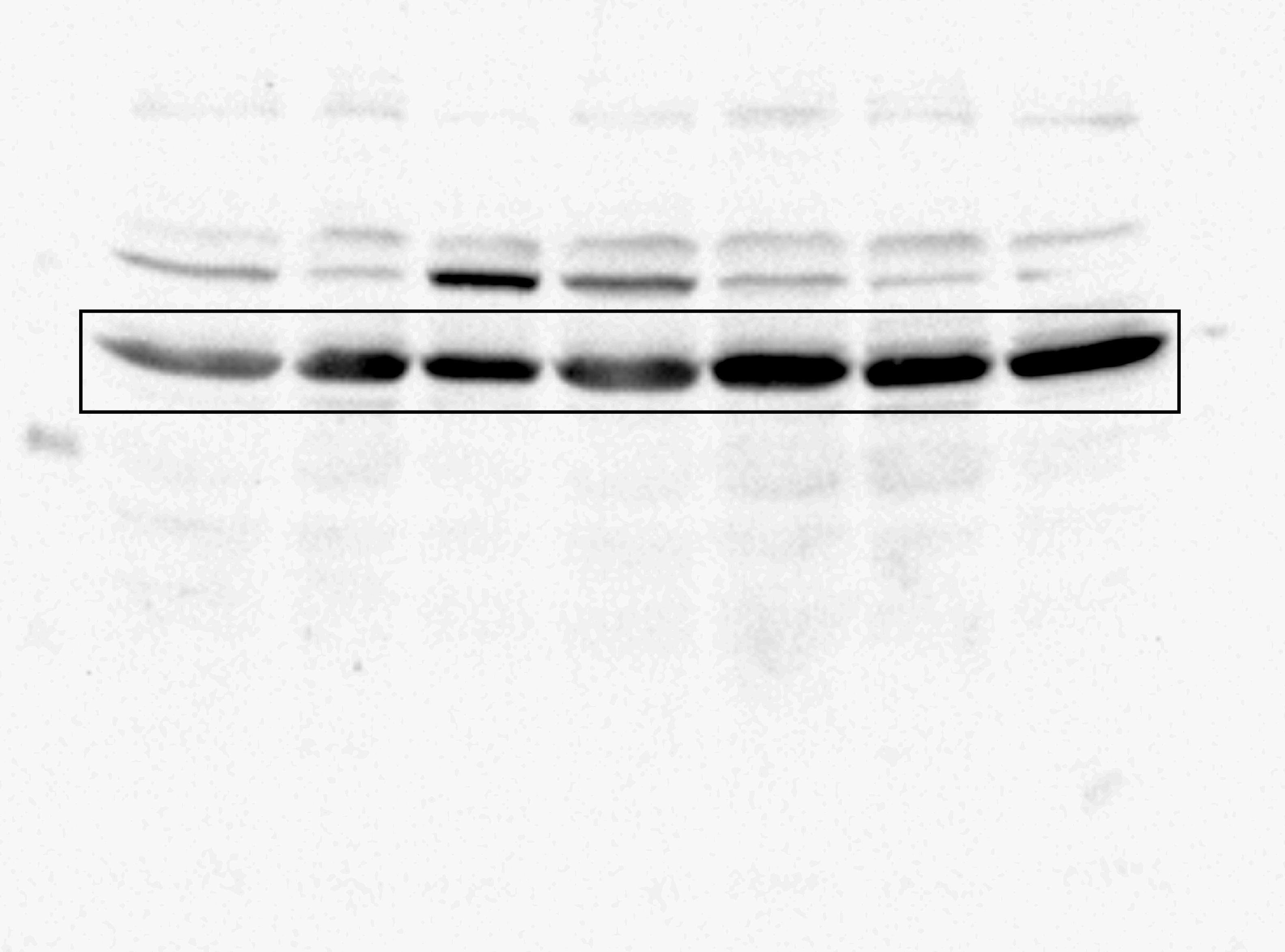

Supplement: Supplemental Information 3 — The complete membrane from which the middle panel of Fig. 5A was taken. [file peerj-07-7494-s003.png]

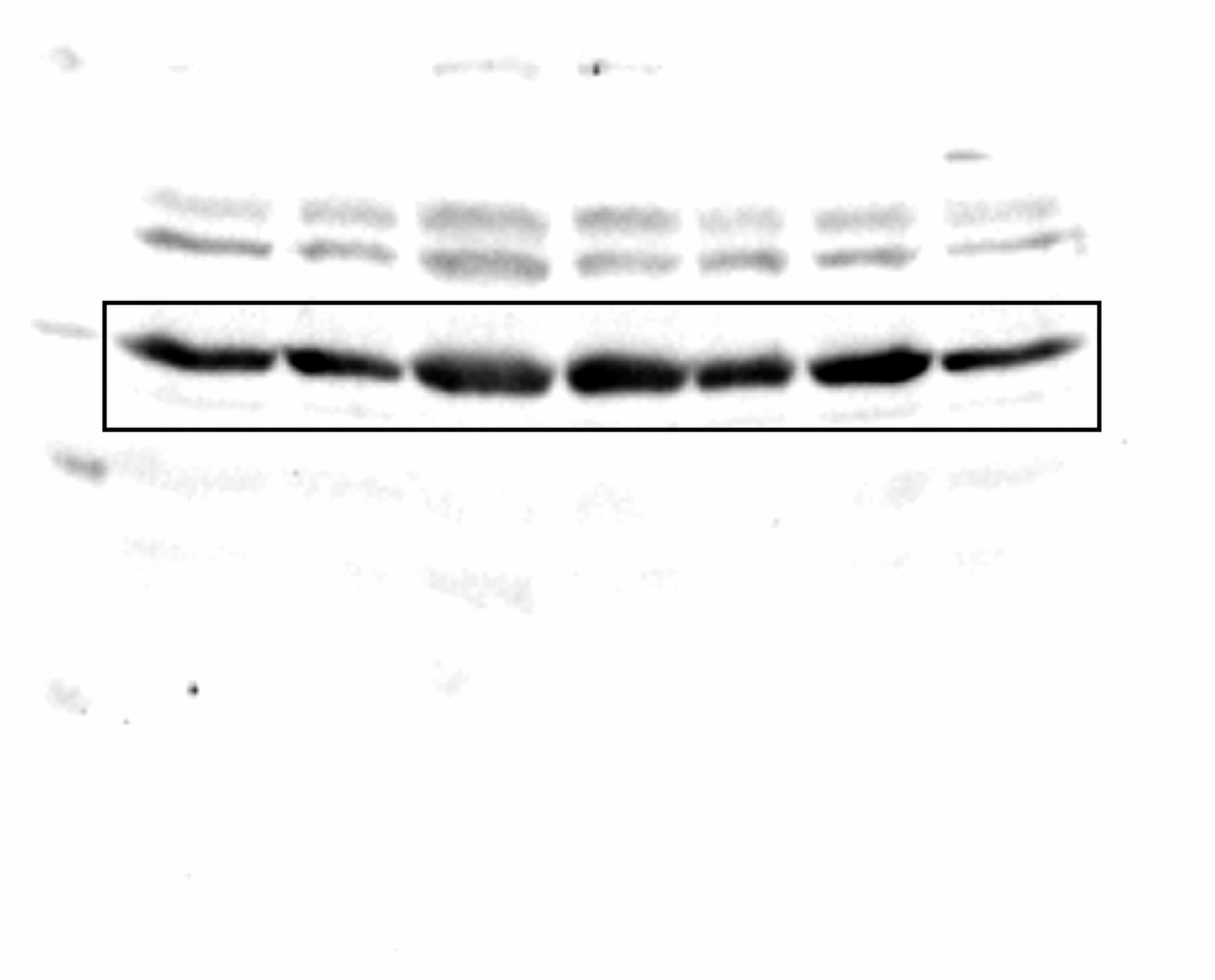

Supplement: Supplemental Information 4 — The complete membrane from an independent experiment corresponding from which the middle panel of Fig. 5A was taken. [file peerj-07-7494-s004.png]

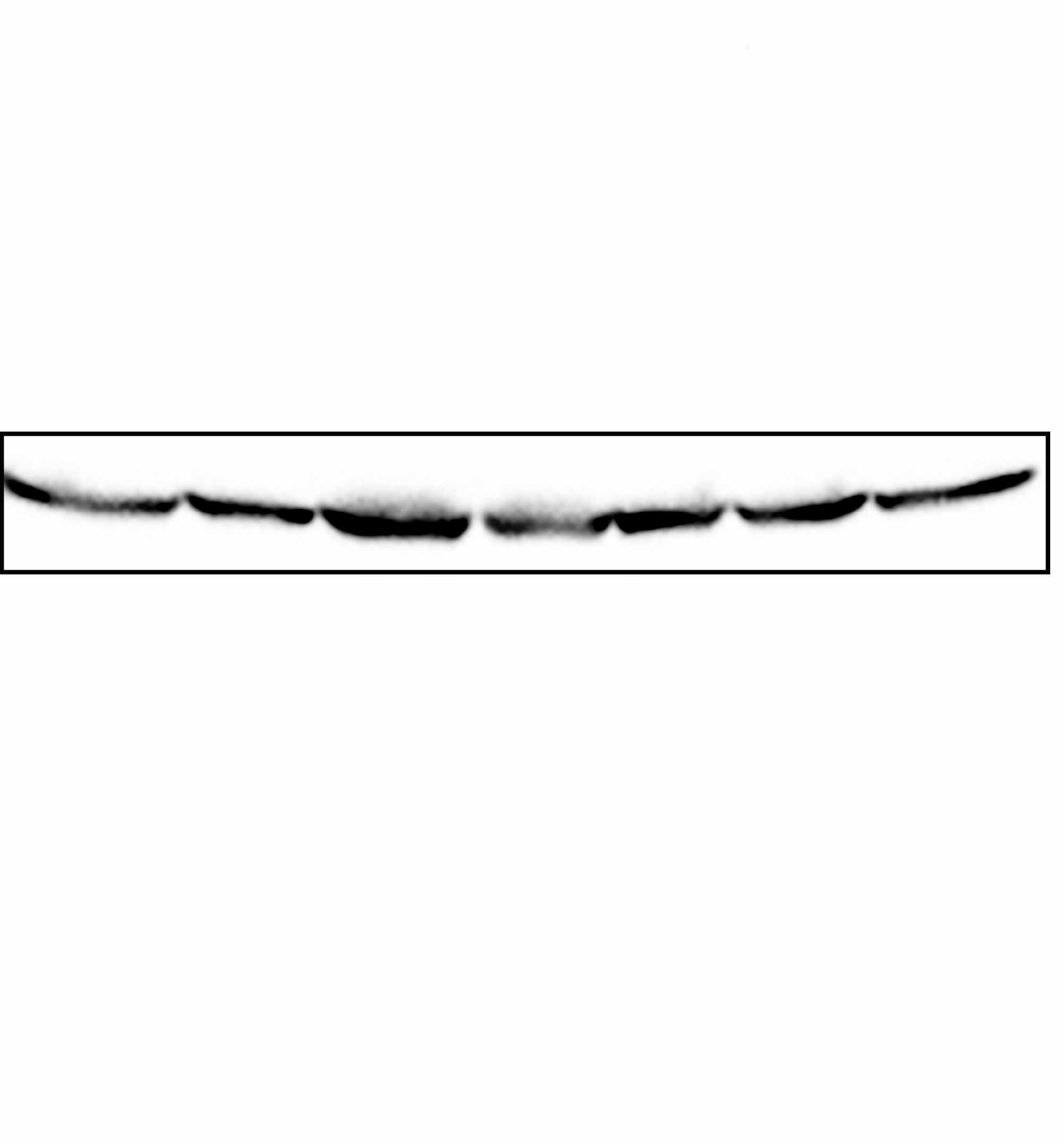

Supplement: Supplemental Information 5 — The complete membrane from which the lower panel of Fig. 5A was taken. [file peerj-07-7494-s005.png]

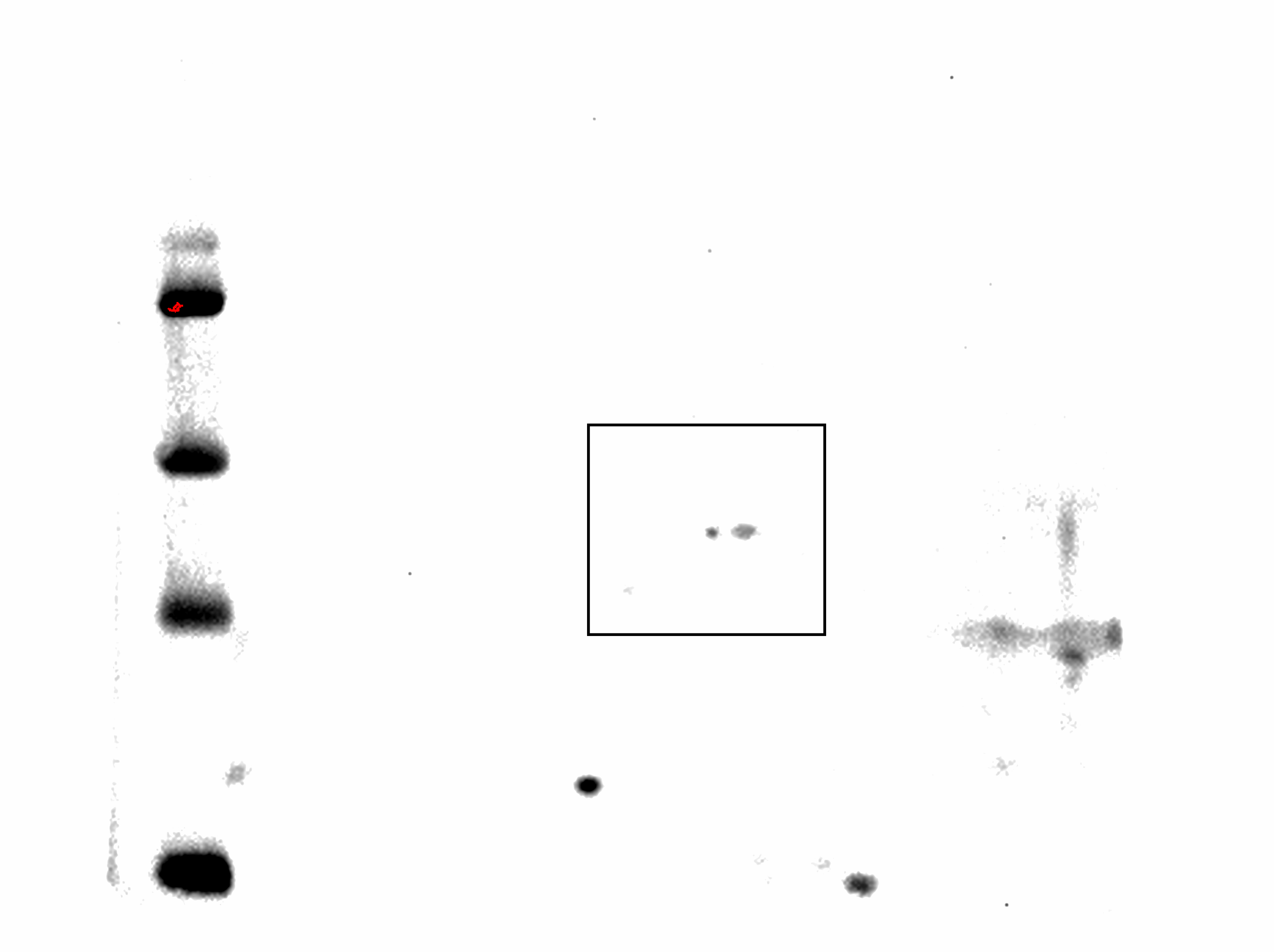

Supplement: Supplemental Information 6 [file peerj-07-7494-s006.png]

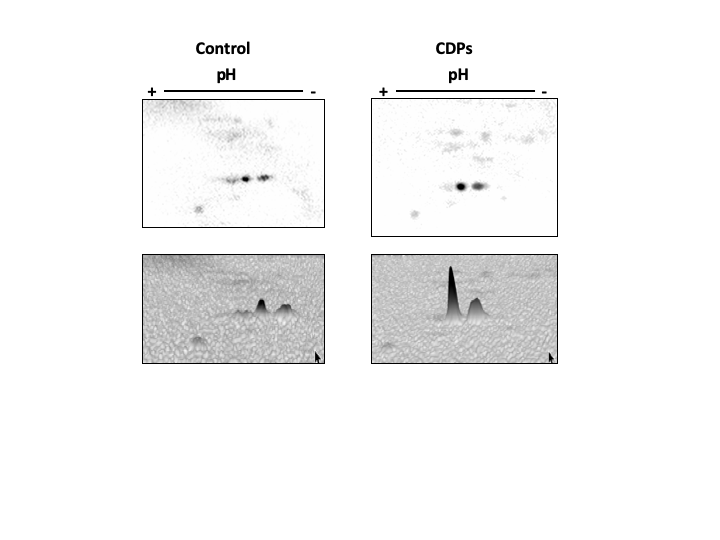

Supplement: Supplemental Information 7 [file peerj-07-7494-s007.png]

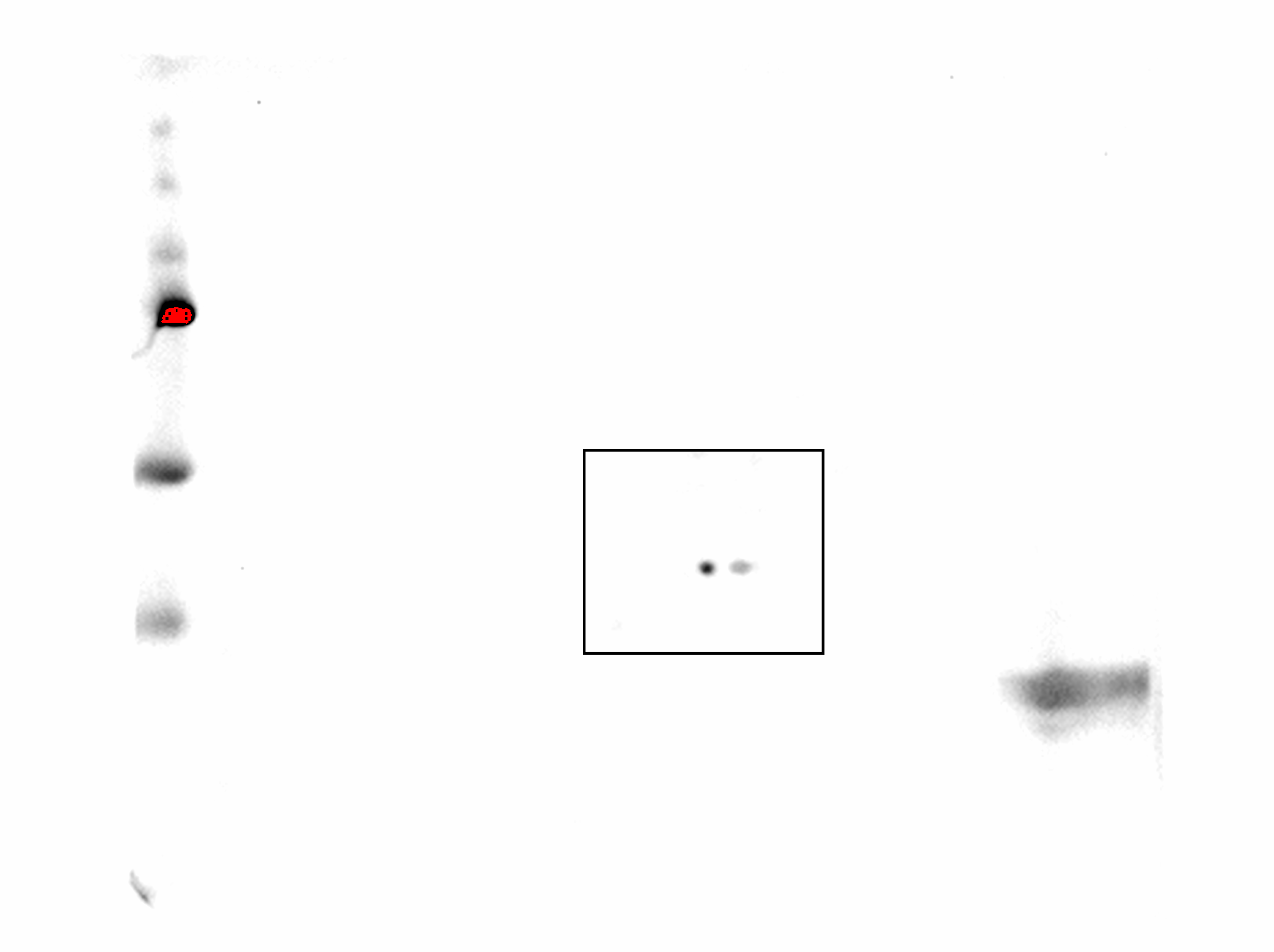

Supplement: Supplemental Information 8 [file peerj-07-7494-s008.png]

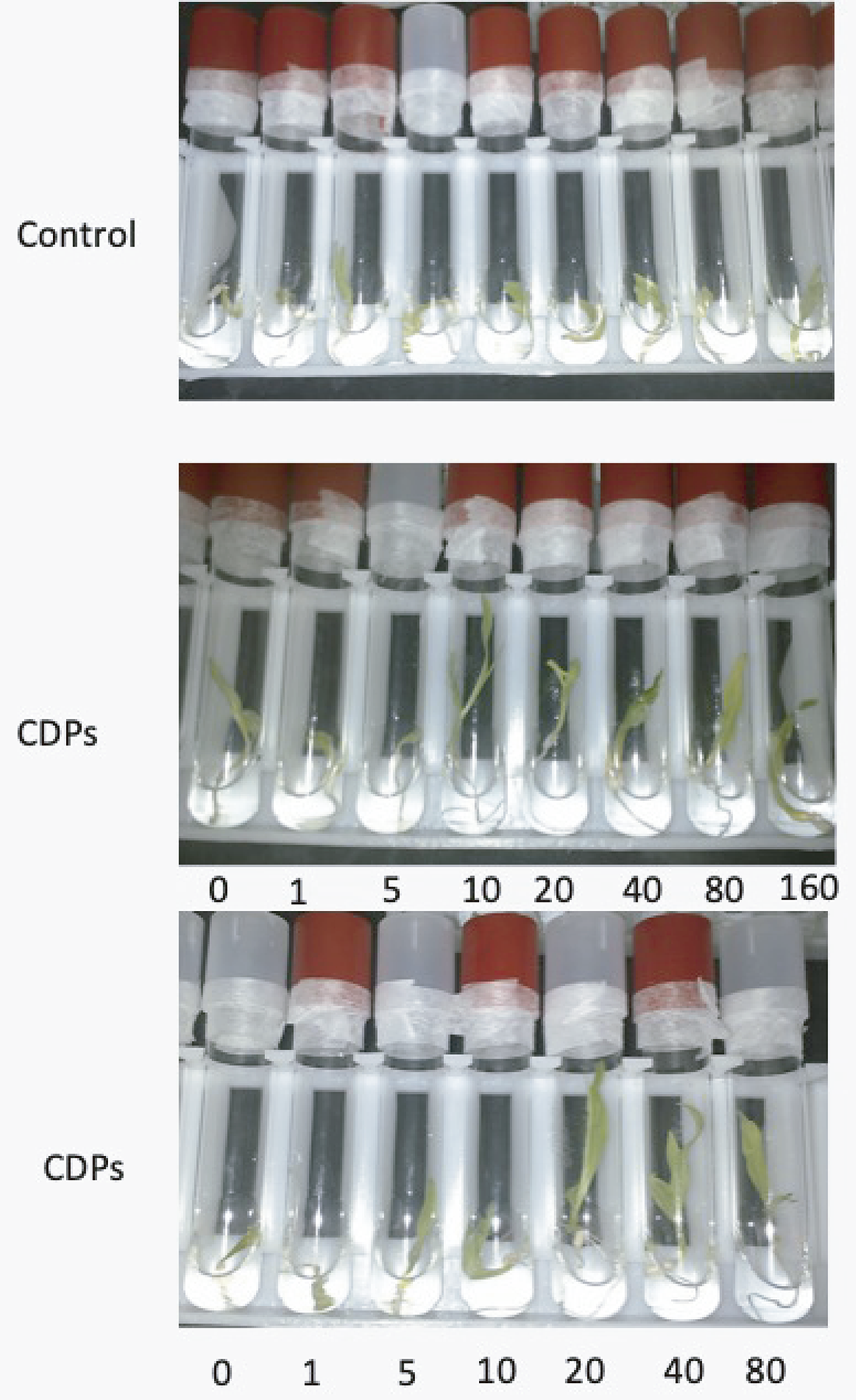

Supplement: Supplemental Information 12 — Photograph of maize plants growing in control conditions and in liquid MS medium supplemented with the indicated concentrations of CPDs (uM). [file peerj-07-7494-s012.png]
